# Editorial: Reviews in psychiatry 2023: aging psychiatry

**DOI:** 10.3389/fpsyt.2025.1601909

**Published:** 2025-04-25

**Authors:** Cristiano Capurso

**Affiliations:** Department of Medical and Surgical Sciences, University of Foggia, Foggia, Italy

**Keywords:** aging, frailty, cognitive impairment, dementia, Alzheimer disease, Levy body dementia

The world’s population is expected to continue to grow over the next 50 to 60 years, peaking at around 10.3 billion people in the mid-2080s, up from 8.2 billion in 2024. By the late 2070s, the number of people aged 65 and over globally is expected to reach 2.2 billion, surpassing the number of people under 18. In fact, by the mid-2030s, there will be 265 million people aged 80 and over, more than the number of people aged 1 year and under (1).

In countries where populations have already peaked or are projected to peak in the coming decades, this has profound implications for health and social care planning and delivery. The most challenging expression of population ageing is the clinical condition of frailty, a state of increased vulnerability to sudden changes in health status triggered by minor stressors, with a resulting increased risk of falls, disability, long-term care, and death (2–7). However, if an estimated 31–51 percent of people aged 80 and over are frail, then 49–69 percent of people aged 80 and over may not be frail (8), raising questions about how frailty develops, how it might be prevented, and how it can be reliably detected.

The aging brain is associated with characteristic structural and physiological changes, particularly involving neurons with high metabolic demands, such as the hippocampal pyramidal neurons, that can be affected disproportionally by changes in synaptic function, protein transport, and mitochondrial function (9).

Brain aging is also characterized by structural and functional changes in microglial cells, which are the resident immune cell population of the central nervous system (CNS) and have a macrophage function. They are activated by both brain injury and local and systemic inflammation and become hyper-reactive to even small stimuli with aging, resulting in neuronal damage and death (10–12).

Growing evidence has supported the hypothesis of a temporal association between frailty, cognitive impairment, and dementia.

In particular, evidence from prospective cohort studies showed an independent association between frailty and dementia, where the association of health status deficits that were among the markers of frailty significantly increased the risk of dementia. More specifically, a higher level of physical frailty was associated with a faster decline in cognitive function until the onset of dementia (13–15).

Aging psychiatry continues to evolve as new research refines our understanding of neurodegenerative conditions, diagnostic methodologies, and patient-centered approaches.

Older adults are still overlooked in clinical trials for several reasons. First, outpatient clinical trials are more difficult to conduct for people aged 85 years or older than for those aged 65–74 years, so subjects in such trials are not representative of the majority of frail older adults. Second, older adults have multiple coexisting illnesses, both physical and psychiatric, which are confounding factors and make it difficult to study pure forms of a disease, such as dementia (16).

On the other hand, the precision medicine (PM) strategy has also determined a major paradigm shift in neuroscience and Alzheimer’s disease (AD) research and development, moving away from the classic “one-size-fits-all”. Progress towards the holistic strategy based on PM systems may translate into a better understanding of the pathogenetic processes of diseases, including dementia, perhaps inaugurating a new era of scientific and therapeutic success of dementia and AD (17).

In this regard, the document “2018 National Institute on Aging—Alzheimer’s Association (NIA-AA) Research Framework” proposes an update to the 2011 guidelines for the diagnosis of AD based on a biological rather than syndromic approach (18). AD is defined by underlying disease processes, documented by postmortem examinations or *in vivo* biomarkers. The framework introduces the AT(N) classification system, which groups biomarkers into three categories: amyloid-beta deposition (A), pathological tau (T), and neurodegeneration or neuronal injury (N). This system is flexible and can be expanded with new biomarkers. The document emphasizes that the framework is intended for observational and interventional research, not routine clinical practice, and aims to create a common language for investigators to generate and test hypotheses about the interactions between disease processes and cognitive symptoms.

The Dementia with Lewy Bodies (DLB) Consortium also refined its recommendations for the clinical and pathological diagnosis of DLB in the Fourth Consensus Report (19). It updates previous recommendations by clearly distinguishing between clinical features and diagnostic biomarkers and providing guidance on the optimal methods for establishing and interpreting them.

Key updates include:

- Increased diagnostic weight given to REM sleep behavior disorder and 123iodine-MIBG myocardial scintigraphy.
- Description of the diagnostic role of other neuroimaging, electrophysiological, and laboratory tests.
- Minor changes to pathological methods to account for the neuropathological changes of Alzheimer’s disease and to include pathological categories related to Lewy bodies.

Key updates to diagnostic criteria include:

- Definition of dementia: It is defined as a progressive cognitive decline that interferes with social or occupational functioning.
- Main clinical features: cognitive fluctuations, recurrent visual hallucinations, REM sleep behavior disorder, and parkinsonism.
- Supportive clinical features and indicative and supportive biomarkers.

Finally, recommendations on clinical management are provided, based mainly on expert opinion, given the paucity of randomized controlled trials, i.e., the importance of a multifactorial approach that includes thorough initial assessment, early identification of symptoms, education and support for caregivers, and pharmacological and non-pharmacological interventions.

The document emphasizes the need to better understand the neurobiology and pathophysiology of DLB, develop and conduct clinical trials, and provide adequate information and support to patients and caregivers.

The main objective of this Research Topic was to provide critical insights into DLB, AD diagnosis, PM, and the impact of frailty on stroke outcomes. Together, they highlight the complexity of cognitive deficits in older populations and underscore the need for multidisciplinary strategies in both research and clinical practice, including identifying strategies to delay the pathological effects of aging.

Bussè et al. provide a comprehensive review of the cognitive markers distinguishing DLB from AD, emphasizing the prominence of visuospatial and executive dysfunctions in the early stages of DLB.

In their review they provide evidence that a cognitive profile characterized by substantial impairments in visual cognitive abilities and relatively better performance on memory tasks that depend on hippocampal function characterizes the prodromal stage of DLB.

Their work reiterates the necessity of refining diagnostic tools to enhance early identification and differentiation of these neurodegenerative conditions. Given the frequent misdiagnosis of DLB as AD in clinical settings, their findings reinforce the call for targeted cognitive assessments, particularly emphasizing visual-spatial impairment as a diagnostic hallmark.

Xu et al. systematically evaluate the diagnostic potential of Raman spectroscopy for AD, reporting high sensitivity (0.86) and specificity (0.87). Although this research demonstrated that Raman spectroscopy was an effective and accurate tool for diagnosing AD, the authors acknowledge the limitations of their study, primarily the small number of articles included in the review, and also acknowledge the need for further validation before widespread clinical adoption, as the possibility of missed or misdiagnosis cannot yet be ruled out. However, this emerging biomarker technology could complement existing imaging and cerebrospinal fluid assessments, potentially providing a noninvasive and rapid diagnostic alternative.

Siafarikas discusses the growing relevance of PM in aging psychiatry, particularly concerning AD management. The author discusses the application of PM in geriatric psychiatry and AD, where PM aims to provide the right treatment to the right person at the right time, using clinical, genetic, and environmental characteristics to stratify patients and identify targets for therapy.

The review explores various “omics” levels (genomics, epigenomics, proteomics, metabolomics, microbiomics) to better understand the complexity of AD and improve treatment. The need for further research, and individualized treatment regimens as future directions in the field and the importance of a multidisciplinary and personalized approach to delay the onset of dementia and improve the quality of life of older adults are highlighted. However, the author points out that despite theoretical advances, the translation of these principles into routine clinical practice remains limited. The challenge lies in integrating PM within existing healthcare frameworks while addressing ethical and economic concerns.

Li et al. present a systematic review and meta-analysis assessing the role of frailty in post-stroke outcomes. The paper involved 25 studies with a total sample of 90,118 patients. Their findings suggest that frailty significantly increases the risk of mortality and poor functional recovery. Specifically, the prevalence of frailty among stroke patients was found to be 23%. Furthermore, after adjusting for potential confounders, their analysis showed that frailty in stroke patients was associated with 1.22 times higher odds of mortality compared to stroke patients without frailty. The authors emphasize the importance of including frailty assessment in stroke care pathways, underscoring the need for preventive interventions to mitigate adverse outcomes and improve quality of life. This is in line, the authors conclude, with broader discussions on holistic patient management, advocating for a more comprehensive approach that includes the cognitive, physical, and social dimensions of aging.

In conclusion, the studies presented in this Research Topic, as well as in the wider scientific literature in general, collectively underscore the importance of interdisciplinary research in aging psychiatry. From refining differential diagnoses in dementia to advancing biomarker-based diagnostics and implementing personalized medicine, the field is poised for transformative progress. Further research should focus on large-scale validation studies, real-world implementation of precision medicine, and integrated care models that account for frailty as a determinant of health outcomes. As the global population ages, the demand for refined, evidence-based strategies in psychiatric and neurological care will only intensify.

We hope that the papers presented in these Research Topics will contribute to raising awareness of geriatric psychiatry and its constant evolution from a “static” discipline, i.e. one primarily concerned with the care of frail elderly people, to a more “dynamic” discipline, which uses the knowledge acquired from studies on psychiatric research and treatment on aging to develop new therapies for brain disorders of late life and extend the effective health span of the elderly, changing the paradigm of studies from “geriatric psychiatry” to “brain aging and mental health” (20).

By critically examining these recent contributions, we can better shape future research priorities and clinical interventions, ensuring that aging individuals receive the most accurate diagnoses, effective treatments, and comprehensive care.
